# Estimation of milkability breeding values and variance components for Italian Holstein

**DOI:** 10.3168/jdsc.2021-0167

**Published:** 2022-03-03

**Authors:** Ferdinando Galluzzo, Jan-Thijs van Kaam, Raffaella Finocchiaro, Maurizio Marusi, Shogo Tsuruta, Martino Cassandro

**Affiliations:** 1 Associazione Nazionale Allevatori della Razza Frisona, Bruna e Jersey Italiana (ANAFIBJ), Via Bergamo 192, 26100 Cremona (CR), Italy; 2 Department of Animal and Dairy Science, University of Georgia, Athens 30602; 3 Department of Agronomy, Food, Natural resources, Animals and Environment (DAFNAE), University of Padova, Viale dell'Università 16, 35020 Legnaro (PD), Italy

## Abstract

The importance of milkability as a trait is growing because of the need to efficiently use labor and machinery; therefore, it is crucial to update the statistical model for the trait to improve the accuracy of the estimated breeding values, and thus provide a more accurate tool for decision-making at the farm level. In the Italian Holstein Friesian cattle population, milkability is recorded twice a year by the milk recording system as a binary trait (slow, coded as 2, or not slow, coded as 1). Data consisted of 7,862,371 records from 2,945,249 cows collected between 2004 and 2021. A single-trait threshold animal model with repeated measures was used, with parity, days in milk class, calving season, and regression of production (fat + protein grams) within days in milk class as fixed effects and herd-year-season of recording, permanent environment, and animal as random effects. The results for heritability and repeatability were 0.275 and 0.5, estimated with the Gibbs sampler THRGIBBS1F90. Genomic validation, carried out using genotyped proven bulls born before 2009 as the training set, gave a result of 0.386 for reliability. The genetic correlations of this trait confirmed that both extremes of the estimated breeding value must be treated cautiously, because correlations with important traits such as mastitis resistance, body condition score, and teat length are unfavorable.

## Highlights

• The national genetic evaluation for milkability in Italian Holstein was revised.
• A stricter data editing and a different approach to the phenotype, from ratio to single observations, were applied.
• A different statistical model was used, changing from a linear to a threshold model.
• Higher genomic reliability was achieved with the revised model.
• The revised model provides more reliable breeding values for decision-making at the farm level.

## Graphical Abstract

**Figure:**
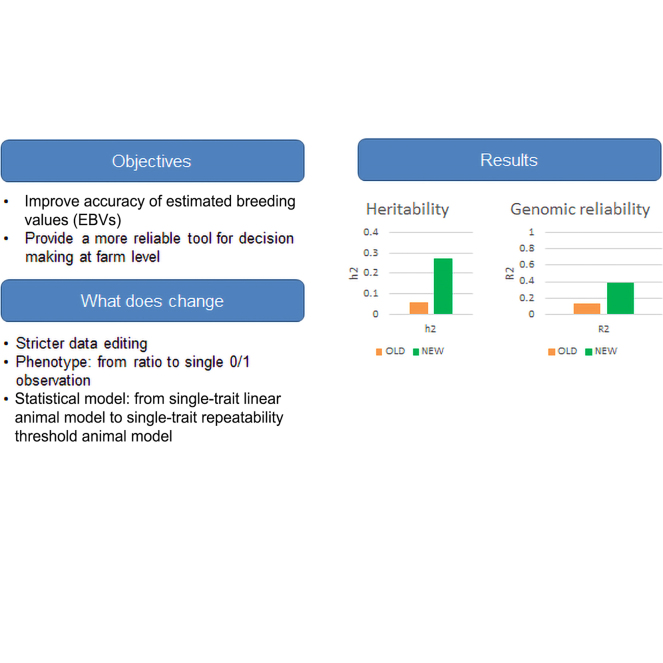
**Summary:** The importance of milking speed is growing due to the need for efficiency at the farm level; therefore, it is important to update and improve the statistical model for the estimation of breeding values for this trait. A strict data editing procedure was applied. The defined model was a single-trait repeatability threshold animal model. The results for heritability and repeatability were 0.275 and 0.5. The result of the genomic validation for reliability was 0.386, which is an improvement compared with the previous model. The genetic correlations of this trait confirmed that both extremes of the estimated breeding value must be treated cautiously.

Milkability is the ability to secrete milk in a regular, complete, and fast way: it can be defined as “workability,” as it is regarded as a management trait. The importance of this trait in dairy farms is currently increasing because of the need for cost-efficient use of labor and machinery caused by the lowering of profit margins as a result of fluctuations in milk and feedstuff prices. Milkability is a trait in which extreme high and low values must be handled carefully; in fact, although the time requirements of slower cows are a management issue, high milking speed is genetically correlated with a higher SCS and a higher incidence of udder infections (Emanuelson et al., 1988; Rupp and Boichard, 1999; Govignon-Gion et al., 2016; Marete et al., 2018). From a practical standpoint, milkability breeding values can be used to optimize individual mating or as a threshold trait in bull selection when milking practices require minimizing the presence of slow cows in the herd.

In Italy, milkability is recorded twice a year as a binary trait (slow, coded as 2; or not slow, coded as 1) by the milk recording system, resulting in nearly 2 observations per cow per parity. The observation refers to the subjective score of the farmer. The aim of this study was to update the statistical model on which our milkability breeding values for Italian Holstein are based and estimate the genetic parameters and correlations with other EBVs. This will lead to more efficient use of the huge amount of data coming from the milk recording system and to an improvement in the accuracy of EBVs, with the aim of providing a more accurate tool for decision-making at the farm level. The analyzed data included records from 2004 onward. This study did not involve animals and prior ethical approval was deemed not necessary.

The previous model was a linear model in which the observations were the ratios between the number of controls in which an animal was recorded as slow and the total number of records for that animal. The fixed effects were parity, milk yield in kilograms on the day of recording, and contemporary groups of herd-year-season (**HYS**) of recording (January–June or July–December). As the number of observations per animal was 1 but the number of recordings per animal was >1, we must define precisely each of the fixed effects. Parity referred to the parity of an animal at the last recording if the ratio was 0, or its parity on the last occasion when it was recorded as “slow.” The random effects were the animal genetic effect and the residuals.

The new model included recorded parity, season of calving, HYS, DIM, and milk protein + fat yield at the day of recording. Herds with a frequency of slow cows below 1% or above 30% were excluded from the data set. Regarding milk protein + fat production, outliers were defined using the interquartile range method. In brief, the interquartile range is the difference between the first and third quartiles, and observations outside 1.5 times the interquartile range are considered as outliers.

The minimum number of contemporaries accepted for the HYS variable was 20. The parity variable included 3 classes (1, 2, 3+); season of calving was a binary variable: cold (November–March) and warm (April–October) months; DIM were divided into 7 classes (5–15, 16–30, 31–60, 61–100, 101–150, 151–200, 201–305), and all observations before 5 or after 305 DIM were excluded from the analysis. The pedigree went up to 4 generations. Data consisted of 7,862,371 records from 2,945,249 cows collected between 2004 and 2021. An extensive description of the data set can be found in Table 1.

**Table 1:** Description of the data set

Parameter^1^	Value
Number of observations	7,862,371
Number of animals	2,945,249
Observations/animal	2.67
Number of herds	6,529
Observations/herd	1,204.22
Frequency parity 1	0.46
Frequency parity 2	0.34
Frequency parity 3+	0.21
Number of HYS groups	91,880
Observations/HYS group	85.57
Frequency CalvSea Cold	0.44
Frequency CalvSea Hot	0.56
Frequency DIM1 (5–15 DIM)	0.04
Frequency DIM2 (16–30 DIM)	0.05
Frequency DIM3 (31–60 DIM)	0.11
Frequency DIM4 (61–100 DIM)	0.14
Frequency DIM5 (101–150 DIM)	0.17
Frequency DIM6 (151–200 DIM)	0.16
Frequency DIM7 (201–305 DIM)	0.33
Mean production (g)	2,244.54
SD production (g)	569.87
Records in pedigree	4,213,929
Frequency of slows (mean)	0.029
Frequency of slows (parity 1)	0.031
Frequency of slows (parity 2)	0.028
Frequency of slows (parity 3)	0.033

1 HYS = herd-year-season class; CalvSea = calving season; DIM = days in milk group (1–7); Frequency = proportion of records for the specified effect class (parity, calving season, and DIM group).

Given that applying linear models to categorical traits violates the assumptions of continuity of the outcome and normality—and particularly for genetic parameters estimates, even transformation methods can lead to biased estimations (Abdel-Azim and Berger, 1999)—a single-trait threshold animal model with repeated measures was chosen. The threshold model assumes the existence of an underlying continuous variable that is the sum of several normally distributed fixed and random variables, one of which is the genetic component. The phenotype, slowness in our case, is assumed to be present in those animals in which the variable exceeds the threshold value (Dempster and Lerner, 1950). The chosen model is described below:

*Y_ijklmn_* = *P_i_* + *DIM_j_* + *CS_k_* + b_1_*_j_PROD_ijklmn_* + *hys_l_* + *pe_m_* + *a_m_* + *e_ijklmn_*,

where *Y_ijklmn_* is the underlying liability of slowness; *P_i_* is the fixed effect of parity *i*; *DIM_j_* is the fixed effect of DIM group *j*; *CS_k_* is the fixed effect of season of calving *k*; b_1_*_j_* is the regression coefficient of the linear effect of *PROD_ijklmn_*, in which *PROD_ijklmn_* is the fat + protein yield on the day of recording, within DIM group *j*; *hys_l_* is the random effect of HYS *l*; *pe_m_* is the random permanent environmental (**PE**) effect for cow *m* based on repeated records both within lactation and across parities; *a_m_* is the random additive genetic effect for animal *m*; and *e_ijklmn_* is the residual of observation *n*. The HYS effect was treated as a random effect to avoid extreme-case problems (Misztal et al., 1989).

At the same time, a multiple-trait model was fitted with first and later parities as different traits. For the variance components estimation, the Gibbs sampler THRGIBBS1F90 was used (Misztal et al., 2002; Misztal, 2008) on the entire data set described in Table 1, with 160,000 iterations, a burn-in of 10,000, and a thinning rate of 10 for both models. Convergence was assessed visually.

THRGIBBS1F90 assumes flat priors for fixed effects and noninformative priors for variance components; for binary traits, residual variance is fixed to 1 and threshold to 0 as technical restrictions for model identifiability (Harville and Mee, 1984; Misztal et al., 2002; Chang et al., 2017). As starting values, estimates coming from previous analyses carried out with VCE6 (Groeneveld et al., 2010), treating the dependent variable as linear, were used.

Post-Gibbs analysis was performed using the software POSTGIBBSF90, developed by S. Tsuruta (Aguilar et al., 2018), using the retained 15,000 samples.

As an animal model with repeated measures, heritability was given by

$$h^{2}=\frac{\sigma_{a}^{2}}{\sigma_{a}^{2}+\sigma_{hys}^{2}+\sigma_{pe}^{2}+\sigma_{e}^{2}},$$

where $\sigma_{a}^{2}$ is the additive genetic variance, $\sigma_{hys}^{2}$ is the random effect HYS variance, $\sigma_{pe}^{2}$ is the PE variance, and $\sigma_{e}^{2}$ is the residual variance.

Repeatability (*R*) was given by

$$R=\frac{\sigma_{a}^{2}+\sigma_{pe}^{2}}{\sigma_{a}^{2}+\sigma_{hys}^{2}+\sigma_{pe}^{2}+\sigma_{e}^{2}}.$$

Animals' EBVs were estimated on the same data set used for the estimation of variance components, with the threshold model described above with MiX99 software (Lidauer et al., 2019) and standardized on a scale with mean of 100 and standard deviation of 5: high values mean lower risk of presenting the “slow” phenotype than the average risk within the population.

Genomic validation was done as described in Finocchiaro et al. (2012). Briefly, the animals' EBV were estimated using the MiX99 software (Lidauer et al., 2019) and used to derive estimated deregressed proofs (**EDP**) for 2 data sets: a full data set with all the recorded phenotypes and a reduced data set. Then, genomic evaluation using the SNPblup model was run for the reduced data set to simultaneously estimate the effect of all SNPs, using the EDPs calculated from the reduced data set as response variable. The number of SNPs was 68,263 and imputation was performed with PedImpute software (Nicolazzi et al., 2013). These SNP effects were used to compute the direct genomic values (**DGV**) of 5,504 genotyped bulls with daughters in the full data set but without daughters in the reduced data set (validation bulls). Finally, linear regression was used to regress current EDPs against DGVs of validation bulls: the *r*^2^ value of the linear regression is the reliability of the DGVs of validation bulls. To summarize, the result of the validation process is the reliability of the DGVs of the validation bulls calculated with the reduced data set (when they had no daughters).

Approximate genetic correlations were estimated from the correlations between genomic EBVs and their reliabilities as in Wall et al. (2003), based on the full data set of 387,367 animals with genotypes:

$${\hat{r}}_{g1,2}=\frac{\sqrt{\sum r_{1}^{2}\times \sum r_{2}^{2}}}{\sum \left(r_{1}^{2}\times r_{2}^{2}\right)}\times r_{1,2},$$

with ${\hat{r}}_{g1,2}$ as the approximate genetic correlation between the 2 traits, $r_{1}^{2}$ and $r_{2}^{2}$ as reliabilities of the genomic EBVs for the 2 traits, and $r_{1,2}$ as the correlation between them. Due to the fact that, after 160,000 iterations with 10,000 rounds discarded as burn-in, the multiple-trait analysis gave a very high genetic correlation between first and later parities (0.98), the single-trait model was chosen.

For the single-trait model, post-Gibbs analysis gave a posterior mean of 0.20 [posterior standard deviation (**PSD**): 0.002] for HYS variance, 0.66 (PSD: 0.01) for PE variance, and 0.54 (PSD: 0.008) for additive genetic variance. The effective sample size for each component was >60, with 10 as the recommended minimum (Misztal et al., 2018). Heritability followed a Gaussian distribution and was moderate, with a posterior mean of 0.275 and a PSD of 0.004; the effective sample size was 62.6. Previous studies reported a wide range of values for heritability, between 0.02 and 0.50 (Amin, 2007), depending on the type of data and the statistical analysis. A mean heritability of 0.20 was found for Hungarian Holsteins based on milk flow rates (Amin, 2007), whereas Swedish estimates from repeatability models ranged from 0.24 to 0.43 (Carlström et al., 2014) depending on the trait analyzed (average flow rate, milking time, or box time). For Slovenian Holsteins, heritabilities from 0.03 and 0.25 were reported for subjective scores given by the farmer, depending on the scoring method and the model used (Potočnik et al., 2006). For German Holsteins, values of 0.10 for subjective scoring and values ranging from 0.22 to 0.48 for objective measures were found, depending on the trait analyzed (Dodenhoff et al., 1999; Rensing and Ruten, 2005). In France, values between 0.37 and 0.44 were found (Marete et al., 2018), and in Canada, a mean of 0.14 for Canadian Holstein was reported (Sewalem et al., 2011): both studies were based on subjective scores given by the farmer. In the United States, heritabilities ranging from 0.14 and 0.20 were found for milking duration (Zwald et al., 2005). In Italy, values ranging from 0.19 to 0.24 were found for primiparous Italian Brown, depending on the trait analyzed: total milking time, average milk flow, and its inverse (Povinelli et al., 2003). As the majority of the references are based on different types of data and on linear models, it is difficult to directly compare the results. However, it is possible to divide the literature results in 2 categories: results based on categorical data coming from subjective scores of farmers and results based on continuous data coming from milking system recordings. Regarding the former, literature results range from 0.03 to 0.44, showing high variability, which may be due to the intrinsic bias of subjective scores. For the latter, heritabilities range from 0.14 to 0.48. A comparison of the 2 groups shows that objective measures generally result in higher heritability and less variation of estimates for heritability across countries and populations. The estimate from the present study falls approximately in the middle of the range of both categories: it is higher than most of the results of studies based on subjective scores and lower than those based on objective measures.

Repeatability values found in studies based on objective measures from milking systems, measured as continuous traits, range between 0.47 and 0.89 (Rensing and Ruten, 2005; Gäde et al., 2006; Carlström et al., 2014; Wethal and Heringstad, 2019); for both studies that were based on subjective scores, repeatability was 0.42 (Meyer and Burnside, 1987; Wiggans et al., 2007). Our result was 0.50, which is slightly above the lower bound of the range found in studies based on objective measures and higher than values found in studies based on subjective scores.

Breeding values were estimated using MiX99. The result for the threshold value, expressed on the liability scale ranging from 1 to 2, was 1.94. The solutions for the fixed effects are represented in the graphs in Figure 1 and are expressed on the liability scale. Regarding parity, the solutions show that cows of first and third or later parity are more likely to be recorded as slow compared with second-parity cows. The solutions for calving season show that cows that calve in the cold season are more likely to be slow milkers. The effect of DIM classes clearly follows the milk production curve; for this reason, the fixed regression for production is nested within DIM classes, having a regression coefficient for each class. The result of the genomic validation was a reliability of 0.386 for validation bulls, which is higher than the reliability of the previous model (0.137).

**Figure 1:**
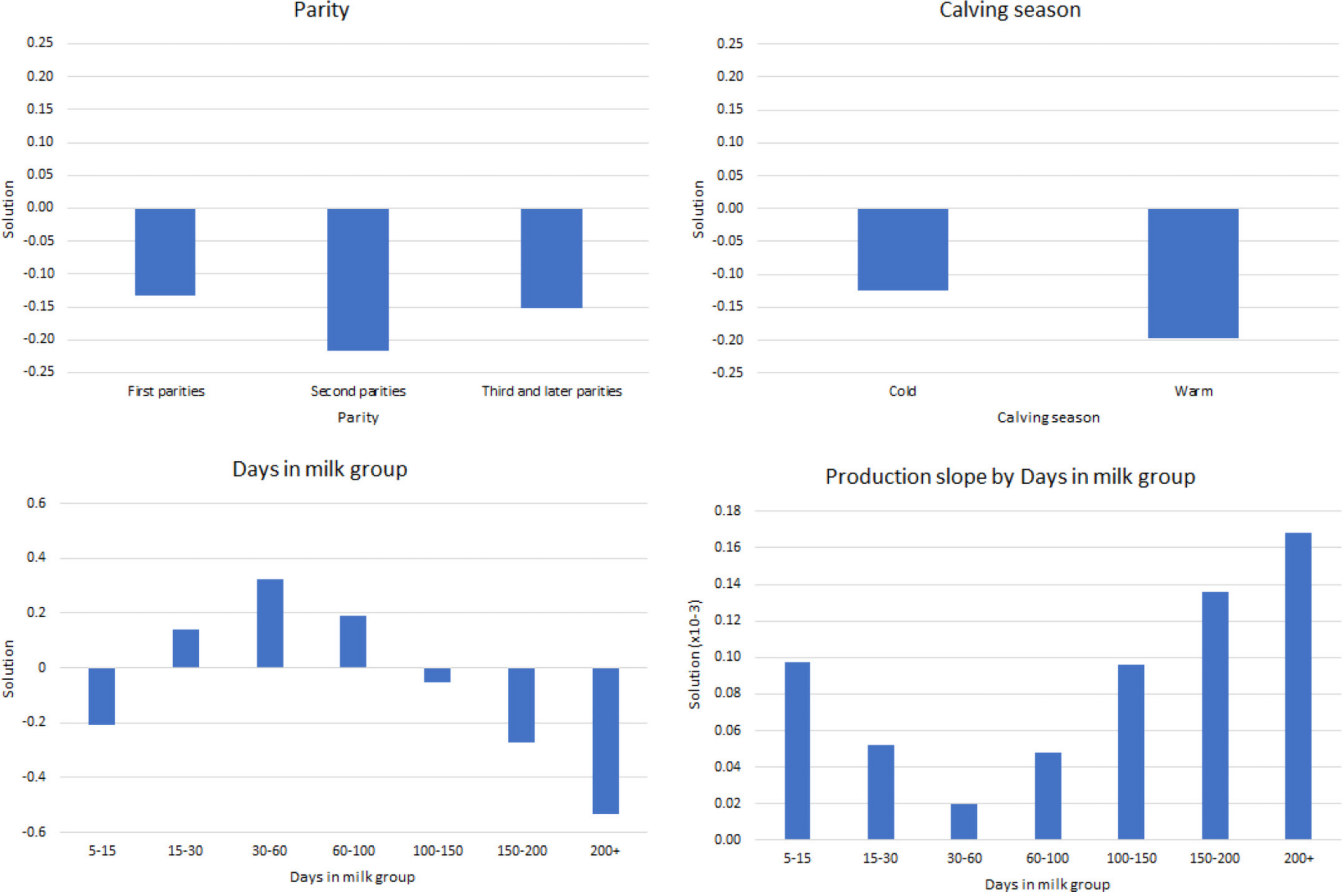
Representation of fixed effects solutions from the MiX99 software (Lidauer et al., 2019) expressed on the liability scale.

The approximate genetic correlations are reported in Figure 2. Regarding production traits, positive correlations with milk (0.14), fat (0.22), and protein (0.18) yields were found. For type traits, milkability was found to be positively correlated with udder traits such as fore udder attachment (0.40), udder depth (0.38), and front teat placement (0.38), meaning that faster cows tend to have a stronger fore udder attachment, a not-too-deep udder, and narrower front teats. These values are higher than those found by Špehar et al. (2017; 0.15, 0.24, 0.20, respectively). A negative correlation of −0.21 was found with teat length, meaning that slower-milking cows have longer teats. This result is in accordance with that of Sewalem et al. (2011) and slightly stronger than the correlation from Špehar et al. (2017; −0.18). Our result is also in accordance with that from Zwald et al. (2005), who found a correlation of 0.20, which was expressed on a reversed scale, meaning that shorter teats are associated with faster milking.

**Figure 2:**
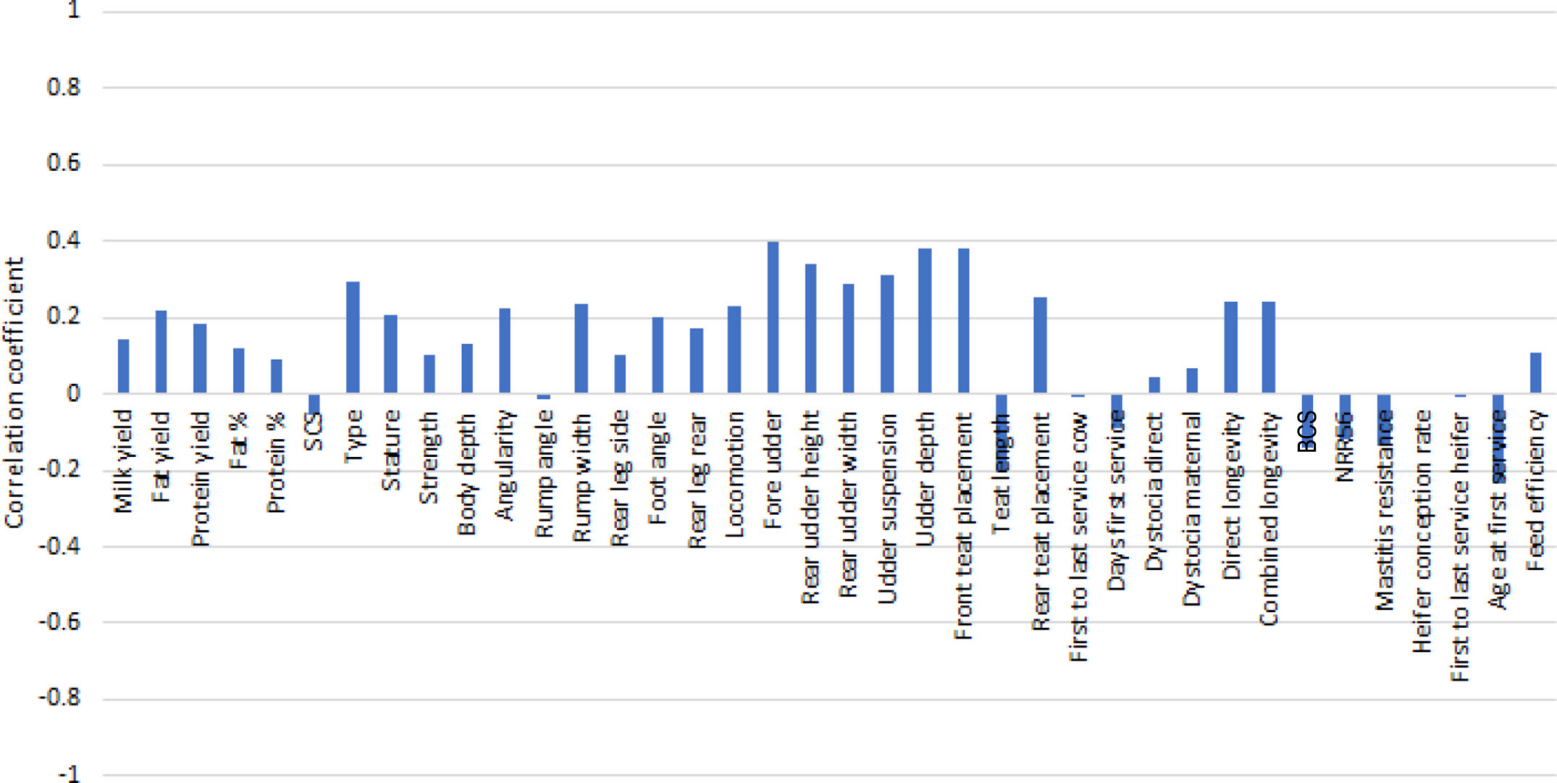
Approximate genetic correlations for milkability EBV. NRR = 56-d nonreturn rate.

Finally, regarding functional traits, a positive correlation with longevity (0.24) was found, different from that (0.03) reported by Rensing and Ruten (2005). Negative correlations were found with BCS (−0.14) and age at first service (−0.24). Regarding udder health traits, we found a weaker than expected and unfavorable negative correlation (−0.06) with SCS. In fact, Rensing and Ruten (2005) and Sewalem et al. (2011) found unfavorable positive correlations between milkability and SCS EBVs of 0.23 and 0.25, respectively, whereas Zwald et al. (2005) found an unfavorable correlation of −0.15 between PTAs of milkability and SCS. Rupp and Boichard (1999) estimated an unfavorable genetic correlation of 0.44 for this pair of traits. In contrast, Potočnik et al. (2006) found a correlation of 0.02 between EBVs for these traits. For mastitis resistance, a stronger negative correlation (−0.14) with milkability was found compared with that between SCS and milkability. This negative correlation (i.e., faster cows have lower resistance to mastitis) was expected and is in accordance with the results from Marete et al. (2018; 0.18) and Govignon-Gion et al. (2016; 0.16). Both of these results indicate that a high milking speed is associated with higher incidence of mastitis. Zwald et al. (2005), in contrast, found a nonsignificant correlation.

Approximate genetic correlations confirm the hypothesis that milkability can be an intermediate-optimum trait because high values are associated with less genetic resistance to mastitis, lower BCS, and shorter and narrow teats, whereas low values are associated with slower milking.

The new model, with a higher genomic reliability (0.386) than the previous model (0.137), increased our ability to estimate the breeding values of animals for this trait, giving farmers a better decision-support tool for their breeding choices. Its genetic correlations, in particular with mastitis resistance (−0.14), indicate that this trait should be handled carefully at its extreme values. Genetic evaluation for milkability can be a tool to lower the financial impact of expensive investments such as automated milking systems by improving the efficiency of the milking routine. Further improvements can be made when additional consistent information from milk flow sensors becomes available.
